# Characterization of intestinal microbiota and fecal cortisol, T3, and IgA in forest musk deer (*Moschus berezovskii*) from birth to weaning

**DOI:** 10.1111/1749-4877.12522

**Published:** 2021-01-22

**Authors:** Yimeng LI, Tianxiang ZHANG, Minghui SHI, Baofeng ZHANG, Xin HU, Shanghua XU, Jianhong DING, Shuqiang LIU, Defu HU, Daniel RUBENSTEIN

**Affiliations:** ^1^ School of Ecology and Nature Conservation Beijing Forestry University Beijing China; ^2^ Department of Ecology and Evolutionary Biology Princeton University Princeton New Jersey USA; ^3^ Beijing Key Laboratory of Captive Wildlife Technology Beijing Zoo Beijing China

**Keywords:** forest musk deer, intestinal microbiota, weaning

## Abstract

Analysis of the intestinal microbiota and physiological parameters in mammalian infancy can reveal health status. In this study, we used a combination of molecular and immunochemical approaches to assess fecal microbiota as well as Cortisol (Cor), Triiodothyronine (T3), and immunoglobulin A (IgA) levels of young forest musk deer (FMD), from birth to one month after weaning (7 days of age–110 days of age). During development as the diet of FMD changes from consuming milk to eating plants, the richness and diversity of intestinal microbiota of young FMD increased significantly. Cor levels remained unchanged throughout early development while significantly increased after weaning, T3 and IgA initially were derived from milk during lactation, significantly decreased after weaning. Correlation network analysis showed that the community of food‐oriented microbes were highly structured and that many genera were correlated. Overall, this study provides scientific insights into effective management strategies for the protection of FMD population.

## Graphical abstract

Analysis of the intestinal microbiota and physiological parameters in mammalian infancy can reveal health status. In this study, we used a combination of molecular and immunochemical approaches to assess fecal microbiota as well as Cortisol (Cor), Triiodothyronine (T3), and immunoglobulin A (IgA) levels of young forest musk deer (FMD), from birth to 1 month after weaning (7 days of age–110 days of age). During development as the diet of FMD changes from consuming milk to eating plants, the richness and diversity of intestinal microbiota of young FMD increased significantly. Cor levels remained unchanged throughout early development while significantly increased after weaning, T3 and IgA initially were derived from milk during lactation, significantly decreased after weaning. Correlation network analysis showed that the community of food‐oriented microbes were highly structured and that many genera were correlated.

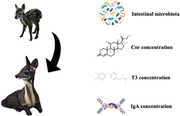

## INTRODUCTION

The mammalian intestinal tract is colonized by large numbers of microorganisms, which engage in a mutually beneficial symbiotic relationship with the host (Backhed *et al*. 2004; Turnbaugh *et al*. 2006; Flint *et al*. 2008; Round & Mazmanian 2009; Walter *et al*. 2011). Intestinal microbiota play an important role in a host's life. These important microbe–host relationships begin with interactions between infants, their mothers and their surrounding physical environments (Knight & Girling 2003; Koenig *et al*. 2011; Isaacson & Kim 2012). Early development of a microbiome impacts the health status and disease occurrence throughout life (Martino *et al*. 2008; Matamoros *et al*. 2013; Aagaard *et al*. 2014). Vael *et al*. (2011), for example, studied 138 human infants and found that gut microbiota with high abundance of *Bacteroides fragilis* and low abundance of *Staphylococcus* between 3 weeks of age and 1 year of age were correlated with increased risk of obesity during later preschool periods. Further, microorganisms are generally considered to be critical for maintaining the metabolism and immune system development of the host in early life (Hooper *et al*. 2012; Cockburn *et al*. 2016). The establishment of intestinal microbiota in animals is a complex process affected by bacteria and host interactions, as well as external and internal factors, such as mode of birth, dietary intake and antibiotic exposure (Flint 2012; Chung *et al*. 2012; Stinson *et al*. 2017). Currently, little research has explored the colonization of intestinal microbiota and the dynamics of some physiological and immune parameters in mammalian young, which hampers the understanding of the physiology dynamics in host early life stage.

Forest musk deer (*Moschus berezovskii*) (FMD), a small forest‐dwelling species unique to Asia, was once widely distributed in the mountainous forest areas of southwestern China. Because of overhunting and habitat loss, the population of wild FMD has sharply decreased since the 1970s. In 2002, China listed the FMD as the key protected Grade I species, and IUCN Red List assessed FMD as an endangered species (Wang & Harris 2015). In 1958, captive breeding of FMD began in China in order to prevent depletion of wild populations. FMD are an easily stressed species, and the incidence of intestinal diseases (diarrhea, enteritis, etc.) led to an increased mortality rate of captive FMD, slowing the growth of the breeding population (Wu & Wang 2006; Sheng & Liu 2007). Simultaneously, according to the wildlife protection plan formulated by the Chinese government, breeding of FMD should precede reintroduction into the natural environment. Therefore, characterizing the establishment of intestinal microbiota as well as some important physiological and immune parameters in newborn FMD will provide a scientific basis for promoting development of captive FMD, benefiting animal welfare and endangered species protection.

Increasing studies have demonstrated that fecal samples provide new opportunities for non‐invasive analysis since they not only can be used to characterize the intestinal microbial communities within animals, they also can be used to measure physiology and immune conditions. For example, Cortisol (Cor) and Triiodothyronine (T3) are important indicators of stress and metabolism, respectively (Beerda *et al*. 2000; Wasser *et al*. 2010). In addition, immunoglobulin A (IgA) is the primary immunoglobulin type on the surface of the intestinal mucosa and plays a key role against invading pathogens at the intestinal mucosa (Dzunkova *et al*. 2016). Together Cor, T3, IgA could serve as important indicators of animal health. Although the intestinal microbiota of adult FMD have been investigated (Li *et al*. 2017), the establishment of gut microbiota and the dynamics of Cor, T3, IgA in young FMD has not been studied. For this current research, 16S rRNA gene sequencing was applied to monitor the colonization of intestinal microbiota in newborn FMD, from birth to one month after weaning (7 days of age–110 days of age), we monitored the changes in fecal Cor, T3, and IgA during this period of development as well.

## MATERIALS AND METHODS

### Ethics statement

The current research was performed according to the guidelines of the Institute of Animal Care and the Ethics Committee of Beijing Forestry University (Beijing, China). The experimental procedures were reviewed and approved by the Ethics Committee of Beijing Forestry University. The breeders in Jiuyao Forest Musk Deer Farm (Sichuan Province, China) approved the collection of young FMD feces samples.

### Sample collection

Experiments were carried out at the Jiuyao Musk Deer Farm (Qingchuan County, Sichuan Province, China). Fifteen healthy and full‐term newborn FMD (vaginal delivery) were selected. Feces were sampled from May 2018 to September 2018. During this period, all the individuals were healthy and were not given antibiotics or anthelmintic drugs. At the farm, the young FMD consumed their mother's milk for the first month after birth. Thereafter, in addition to milk, the young FMD began to eat mulberry leaves (*Morus alba*) as well as a concentrate feed (mainly comprised of wheat bran, corn and soybean meal). Captive young FMD were weaned at 80 d after birth, by separating them from their mothers. From this point onward, the young were raised in separate individual enclosure and fed solely on mulberry leaves (500 g/day) and feed concentrate (100 g/day). Collected fecal samples were divided into 3 stages according to changes in diet: Stage I (7−10 d after birth; *n* = 6) only milk; Stage II (30−80 d after birth; *n* = 45) milk, leaves and feed concentrate; Stage III (weaning to 30 d after weaning; *n* = 30) leaves and feed concentrate. For the first 30 days, fecal sampling is challenging due to the mother FMD's behavior, she stimulates the sucking young to defecate and licks up the feces to retain her instinct of eliminating the fecal smell which attracts predators in the wild. Moreover, in order to avoid mother FMD abandoning the young due to human odor, the feces of the young FMD were not collected during the first 7 days after birth. The fecal samples of Stage I were collected by professional breeders who swabbed the anus with cotton balls to stimulate the young FMD to defecate. Also, feces were not collected from young FMD showing strong stress responses (e.g. keep tweeting and struggling). Thirty days after birth, the activity of young FMD gradually increased and the feces were excreted onto the ground. The procedure of sampling was performed as follows: The evening before sampling, the enclosure was cleaned thoroughly and fresh feces of young FMD were collected before dawn the following day. Those collecting feces wore disposable surgical sterile gloves and fecal samples were put into sterile centrifuge tubes, sealed, marked and preserved at −80°C.

### DNA extraction, PCR amplification, and sequencing

The Power Soil DNA Isolation Kit (MO BIO Laboratories) was used in this study to extract Bacterial DNA. Spectrophotometry set at wavelengths 260 nm/280 nm & 260 nm/230 nm was used to measure the quality and quantity of extracted DNA. PCR (Tiangen Biotech, Beijing CO., LTD., China) was used to amplify the 16S rRNA gene (V3‐V4 hypervariable region) via the primer pair (338F, 5′‐ACTCCTACGGGAGGCAGCA‐3′; 806R, 5′‐GGACTACHVGGGTWTCTAAT‐3′). The PCR amplification was conducted using an Ultra High‐Fidelity PCR kit. Volumes of 50 μL, contained 10 μL of PCR buffer, 0.2 μL of Q5 High‐Fidelity DNA polymerase, 10 μL of High GC Enhancer, 1 μL dNTP (500 μM), 1.5 μL each of primer pairs (10 μM), 60 ng template DNA, and doubly‐distilled H_2_O. PCR cycling procedure started with denaturation for 5 min at 95°C followed by 15 cycles comprising 1 min at 95°C, 1 min at 50°C, 1 min at 72°C, ending with 7 min at 72°C. VAHTSTM DNA Clean Beads were used for PCR products purification. Next, another PCR amplification was performed in a total 40 μL volume, contained 10 μM of primer pairs, 20 μL of 2 × phusion HF MM, 8 μL of doubly‐distilled H_2_O and 10 μL of previous PCR products. The PCR cycling procedure involved 30 s of denaturation at 98°C, followed by 10 cycles of 10 s at 98°C, 30 s at 65°C, and 30 s at 72°C, ending with 5 min at 72°C. At last, Quant‐iT dsDNA HS kit was applied to do the PCR products quantification. Paired‐end (PE) sequencing was done with the Illumina Hiseq 2500 at Biomarker Technologies Inc., Beijing, China.

### Quantitation of the water content in fecal samples

Fecal samples were milled by mortar to measure water content, fecal hormones (Cor and T3), and IgA levels. Sterile centrifuge tubes were marked G1 with the weight recorded as G1. Feces (≈0.5 g) were then added to the tube, reweighed with the weight recorded as G2. The open tubes were then dried at 65°C in an oven for 8 h until the weight of each tube remained unchanged. This weight was recorded as G3. The feces water content was determined as follows: *P*_water_ = (G2 − G3)/(G2 − G1).

### Determination of fecal hormone levels

A sample of feces weighing 0.50 g was put in 10 mL sterile centrifuge tubes. 5 mL of 90% (v/v) ethanol was added to the tubes, which were agitated for 1 min before being centrifuged at 2500 rpms for 20 min. The supernatant was collected and another 5 mL of 90% (v/v) ethanol was added before being agitated for 20 min and then centrifuged at 2500 rpms for 20 min. The supernatants from the two extraction steps were pooled and the ethanol was evaporated in a 60°C water bath. 1 mL of phosphate buffer solution (PBS) was then added and the tubes were shaken for 5 min using ultrasonic instrumentation. Samples were then frozen at −20°C prior to use. Bovine ELISA kits from Reagent Genie Ltd (Ireland) were used to quantify Fecal Cor and T3 levels because FMD is phylogenetically close to bovids (Hassanin & Douzery 2003; Chen *et al*. 2019). Three replicates were run on each sample.

### Determination of fecal IgA levels

A sample of feces weighing 0.50 g was put into 10 mL sterile centrifuge tubes. 5 mL of phosphate‐buffered saline (PBS) was added and the tubes were agitated for 1 min before being centrifuged at 2500 rpm for 20 min. The supernatants then centrifuged for another 20 min at 10 000 rpm before being frozen at −20°C. Bovine IgA ELISA Quantitation Kit (Bethyl Laboratories, Inc., USA) were used to determine IgA levels. Three replicates were run on each sample.

### Bioinformatic analysis

PRINSEQ was used for the sequence quality control. Only Reads greater than 200‐bp with an average phred quality score greater than 20 were analyzed. Sequences were grouped into operational taxonomic units (OTUs) (97% similarity threshold) using UCLUST v1.2.22 and were compared against the SILVA v132 database (Released on December 13, 2017). The OTUs were then classified into different taxonomic levels using the RDP classifier. QIIME software was used to calculate the Shannon diversity. Non‐metric multidimensional scaling (NMDS) together with One‐way analysis of similarity (ANOSIM) was used to identify differences in bacterial community composition between the 3 developmental stages. Linear discriminant analysis (LDA) effect size (LEfSe) analysis and Phylogenetic investigation of communities by reconstruction of unobserved states (PICRUSt) were conducted using the online Galaxy framework to identify the biomarker in each stage and predict the function of intestinal microbiota. Co‐occurrence network analysis was performed on bacterial genera (top 50 genera relative abundance, Spearman rank *r* > 0.8 or *r* < −0.8 were used) to predict the potential interactions between different species during the 3 stages. Interaction network metrics and plots were analyzed and visualized with Gephi v0.9.2 software based on Fruchterman Reingold layout algorithm. The network consists of nodes and edges (undirected), and clustered using Louvain method (modularity class).

### Statistical analysis

Microsoft Excel 2019 was used to calculate the water content of feces, which was then converted into Cor (ng/g), T3 (ng/g), and IgA (μg/g) values per g of dry feces. The value of each sample was mean of replicates (errors on replicates were <10%). SPSS 22.0 was used for all statistical analyses. Repeated measures one‐way analysis of variance was used to compare the differences in Cor, T3, and IgA levels between different developmental stages. Spearman's correlation coefficient was used to measure the correlation between bacteria and fecal Cor, T3, and IgA levels. Values of *P* < 0.05 and *P* < 0.01 indicated statistically significant and highly significant difference, respectively.

## RESULTS

### Changes of intestinal microbiota richness and diversity in young FMD during growth

Bacterial DNA from feces of young FMD at three stages of growth was extracted and the 16S rDNA hypervariable region (V3–V4) was sequenced. After filtering and screening, 2 626 737 high‐quality sequences (average length 414.69 bp) from 81 fecal samples were obtained. Clustering at 97% similarity level resulted in a total of 831 OTUs.

**Figure 1:**
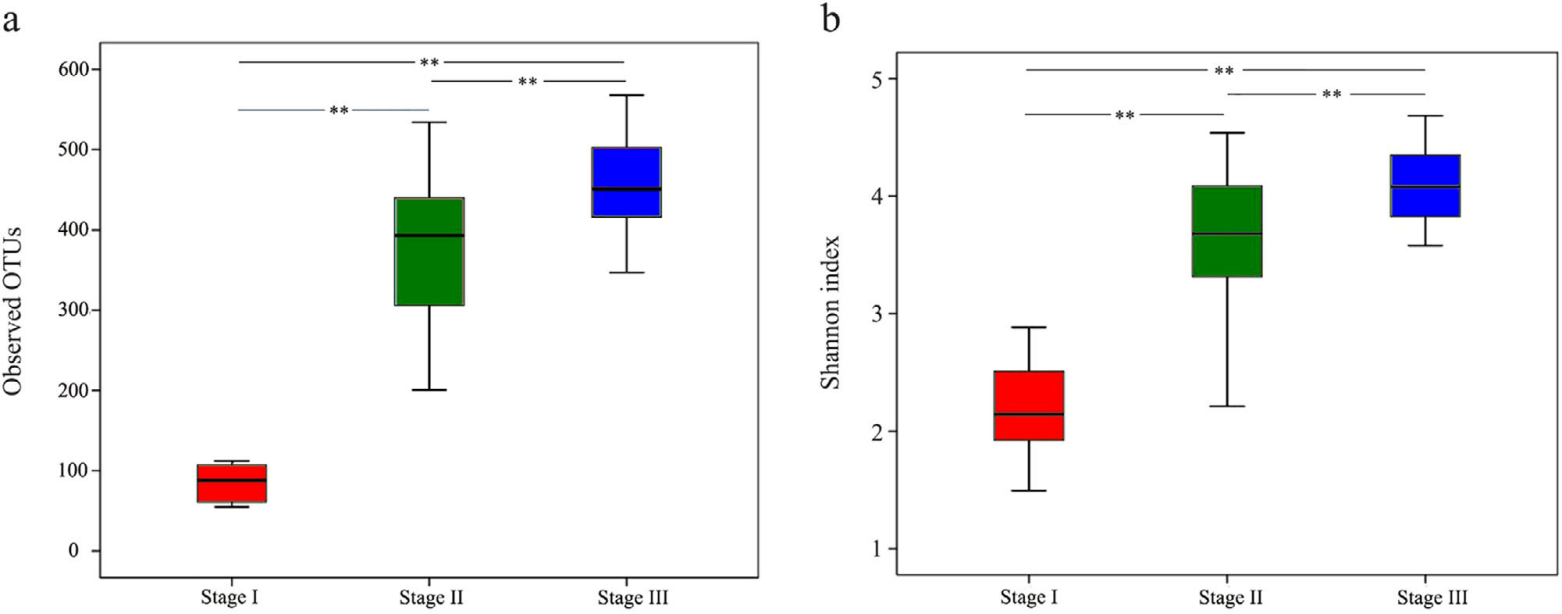
Box plot of diversity indices. (a) OTU richness and (b) Shannon diversity of intestinal microbiota of the young FMD from birth to weaning. Stage I: 7–10 d after birth; Stage II: 30–80 d after birth; Stage III: weaning to 30 d after weaning. Boxes represent the interquartile range (25th to 75th percentiles); and the horizontal line inside the box indicates the median. ^**^ *P* < 0.01 reflects highly significant differences.

Intestinal microbial richness and diversity of young FMD increased significantly over time from Stage I to Stage II to Stage III (Fig. 1a,b). The value of Good's coverage was close to 99% in all samples, suggesting that most bacteria present in the samples were detected.

### Changes of intestinal microbiota composition in young FMD during growth

**Figure 2:**
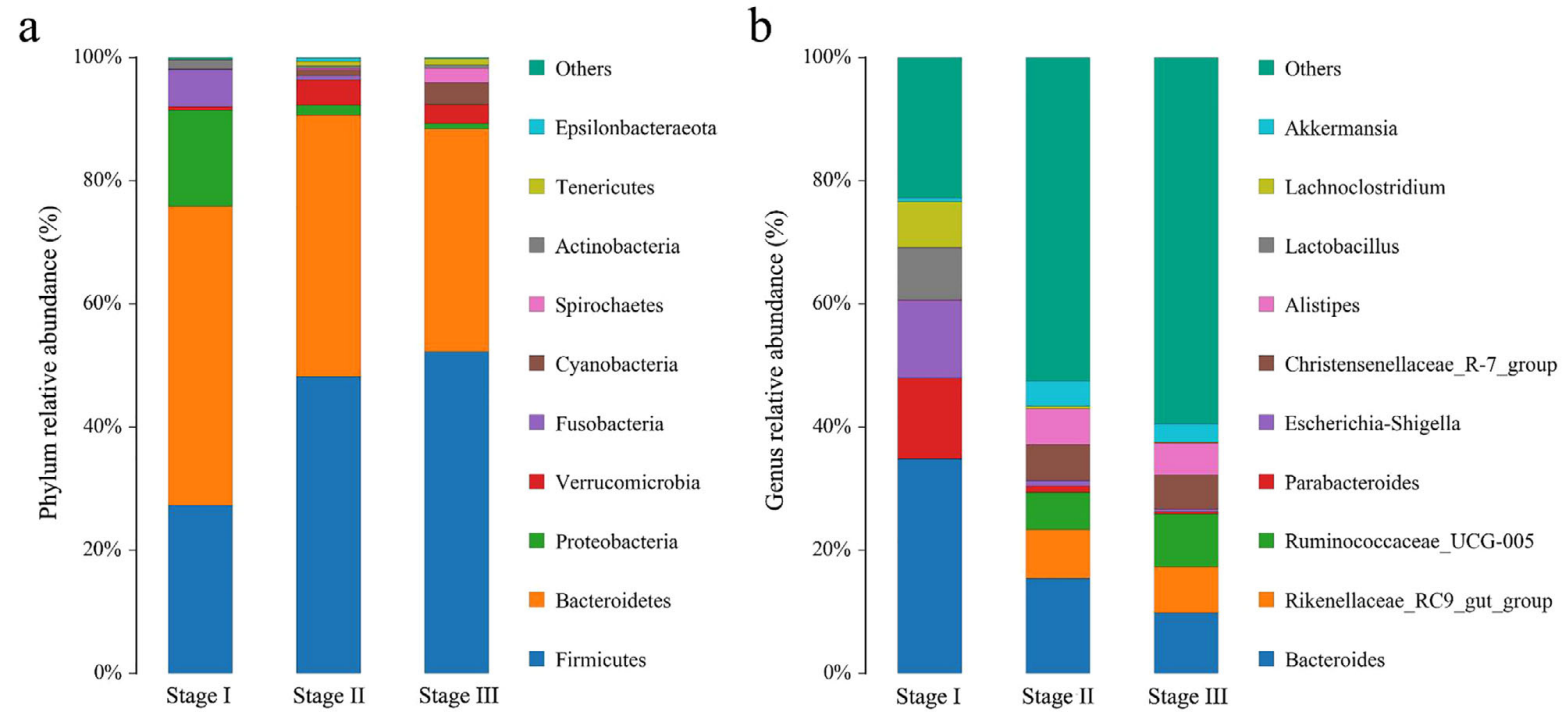
Bar chart of the relative abundance of intestinal microbiota of the young FMD from birth to weaning. (a) Relative abundance (%) of the ten most abundant bacteria phyla. (b) Relative abundance (%) of the ten most abundant bacteria genera. Stage I: 7–10 d after birth; Stage II: 30–80 d after birth; Stage III: weaning to 30 d after weaning. Others: bacteria taxa with abundance ≤1%.

For Stage I, when the young FMD only consumed milk, the main intestinal microbiota at the phylum level were Firmicutes (27.30%), Bacteroidetes (48.51%), Proteobacteria (15.63%), and Fusobacteria (6.14%) (Fig. 2a). At the genus level, *Bacteroides* (34.89%), *Parabacteroides* (13.12%), and *Escherichia_Shigella* (12.54%) were the main intestinal bacteria (Fig. 2b). For Stage II and Stage III, when the young FMD began to eat leaves and feed concentrate, the composition of intestinal microbiota at phylum level and genus level changed from that found in Stage I and became stabilized. At the phylum level, mainly Firmicutes (48.23% in Stage II and 52.23% in Stage III, respectively) and Bacteroidetes (42.43% in Stage II and 36.19% in Stage III, respectively) were detected (Fig. 2a). At the genus level, mainly *Bacteroides* (15.45% in Stage II and 9.89% in Stage III, respectively), *Ruminococcaceae_UCG‐005* (6.09% in Stage II and 8.58% in Stage III, respectively), *Rikenellaceae_RC9_gut_group* (7.87% in Stage II and 7.43% in Stage III, respectively), *Alistipes* (5.78% in Stage II and 5.19% in Stage III, respectively), and *Christensenellaceae_R‐7_group* (5.89% in Stage II and 5.58% in Stage III, respectively) were observed (Fig. 2b).

NMDS plot revealed that Stage I was widely separated from Stage II and III; Stage II and Stage III were clustered (Fig. S1, Supporting Information). ANOSIM analysis showed that microbiota composition had significant differences between Stage I and Stage II (*R* = 0.98, *P* = 0.001), Stage I and Stage III (*R* = 1, *P* = 0.001), while no significant differences between Stage II and Stage III (*R* = 0.02, *P* = 0.29).

### LEfSe analysis and PICRUSt analysis

LEfSe analysis revealed 20 taxa that showed significant differences in abundance in the three diets stages (Fig. S2, Supporting Information). At the phylum level, the abundances of Bacteroidetes and Proteobacteria in the feces of young FMD in Stage I was significantly higher than these in Stage II and III. Firmicutes significantly increased after the young FMD began to consume plant leaves and feed concentrate. At the family level, Lactobacillaceae and Enterobacteriaceae in the feces of young FMD in Stage I were significantly higher than those in Stage II and III, while Prevotellaceae and Ruminococcaceae significantly increased after the young FMD fed on plants and concentrate. PICRUSt analysis shows that the intestinal microbiota has the function of carbohydrate metabolism, amino acid metabolism, lipid metabolism, etc. (Fig. S3a, Supporting Information). When comparing Stage I with Stage II, 3 KEGG pathways showed significant differences, carbohydrate metabolism, metabolism of other amino acids was enriched in Stage I, while energy metabolism was enriched in Stage II (Fig. S3b, Supporting Information). When comparing Stage I with Stage III, carbohydrate metabolism, metabolism of other amino acids and lipid metabolism was enriched in Stage I, while energy metabolism, translation and cell motility was enriched in Stage III (Fig. S3c, Supporting Information).

### Correlation analysis of the top 30 bacterial genera in the intestinal tract of young FMD

**Figure 3:**
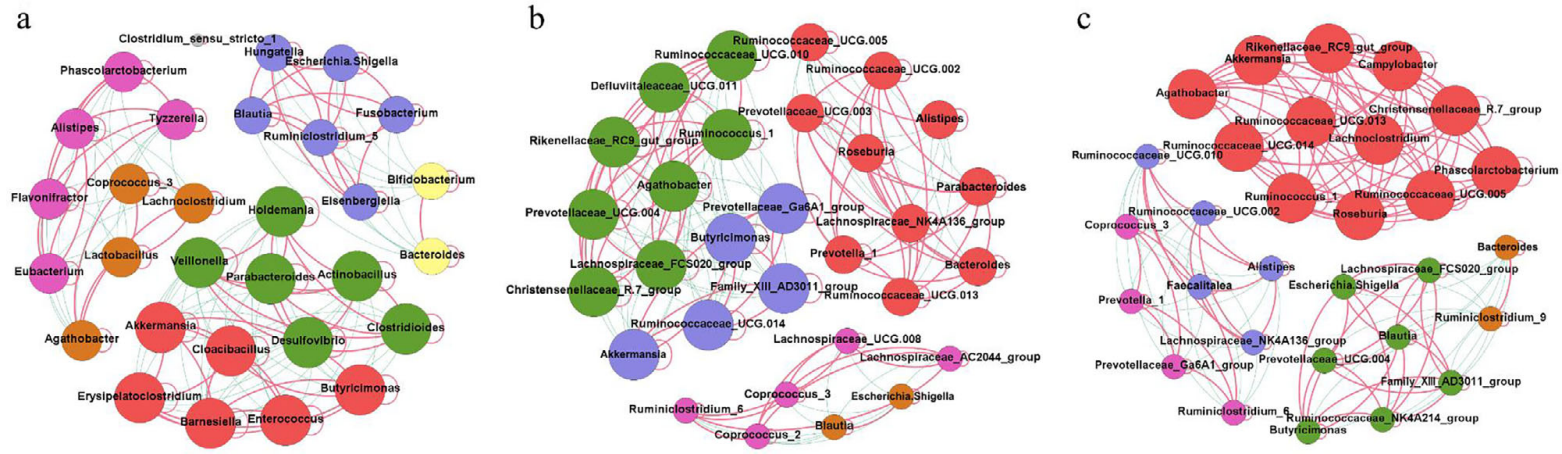
Network analysis of the top 30 bacterial genera in the intestinal tract of young FMD. (a) Correlations of Stage I. (b) Correlations of Stage II. (c) Correlations of Stage III. The network is displayed graphically, as nodes (genera) and edges (biological relationship between the nodes). A connection (i.e. edges) stands for a strong (Spearman rank *r* > 0.8 or *r* < −0.8) and significant (*P* < 0.05) correlation between the genera. The size of a node depends on the number of connections. The same node color indicates genera belong to the same module. A red edge indicates a positive interaction between two individual nodes, while a green edge indicates a negative interaction.

Analysis of the correlation between bacteria can reveal the interaction patterns of coexistence or exclusion, suggesting possible collaborative or competitive relationships between different microorganisms. Accordingly, 3 correlation network graphs were constructed for the top 30 bacterial genera at each stage, based on Spearman correlation coefficient (Fig. 3). In these graphs, genera whose changes in abundance are highly correlated often are clustered into connected modules. In Stage I, the network depicts 7 modules consisting of 160 links between 30 nodes (average degree: 10.667, average path distance: 1.0, average clustering coefficient: 0.953; Fig. 3a); in Stage II, the network depicts 5 modules consisting of 174 links between 30 nodes (average degree: 11.6, average path distance: 1.0, average clustering coefficient: 0.957; Fig. 3b); in Stage III, the network depicts 5 modules consisting of 168 links between 30 nodes (average degree: 11.2, average path distance: 1.0, average clustering coefficient: 0.955; Fig. 3c).

### Changes of fecal Cor levels during the growth of young FMD and correlation with the intestinal microbiota

**Figure 4:**
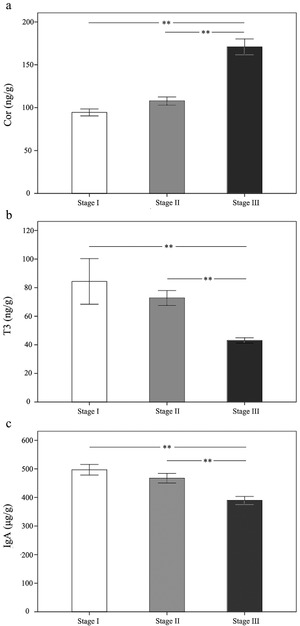
Changes of fecal (a) Cor, (b) T3, and (c) IgA levels in young FMD from birth to weaning. Stage I: 7–10 d after birth (*n* = 6); Stage II: 30–80 d after birth (*n* = 6); Stage III: weaning to 30 d after weaning (*n* = 6). The values represent the mean ± SE. ^**^ *P* < 0.01 reflects highly significant differences.

**Figure 5:**
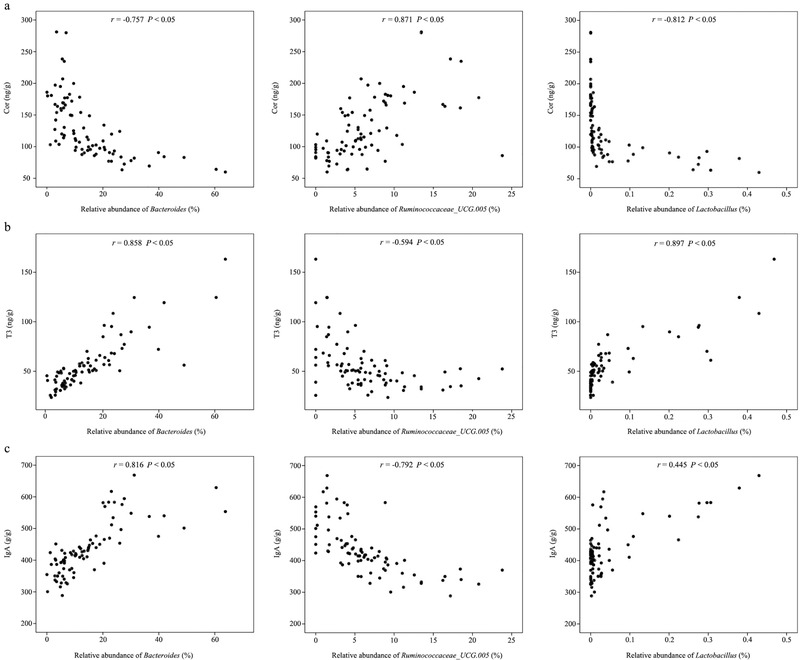
Scatter plot of the relationship between (a) Cor, (b) T3, (c) IgA and bacteria *Bacteroides* (*n* = 81), *Ruminococcaceae_UCG.005* (*n* = 81)*, Lactobacillus* (*n* = 81). The *x*‐axis shows the concentration and *y*‐axis shows the relative abundance of bacteria. Spearman correlation coefficient *r* > 0 and *P* < 0.05 reflects positive correlation, *r* <0 and *P* < 0.05 reflects negative correlation.

During development, Cor levels of young FMD changed significantly (Fig. 4a; *F*_2,10_ = 28.658), but the change was not continuous. From Stage I to Stage II, the fecal Cor levels in young FMD were not significantly different (*P* > 0.05), but Cor levels did increase significantly in Stage III (after weaning) (*P* < 0.01). The scatter plot in Fig. 5a shows that during all the 3 stages, changes in Cor levels were negatively correlated with the change of the abundance of *Bacteroides* (Spearman rank *r* = −0.757, *P <* 0.05), *Lactobacillus* (Spearman rank *r* = −0.812, *P* < 0.05), but positively correlated with the change of *Ruminococcaceae_UCG.005* (Spearman rank *r* = 0.871, *P* < 0.05).

### Changes of fecal T3 levels during the growth of young FMD and correlation with the intestinal microbiota

During development, T3 levels of young FMD changed significantly (Fig. 4b; *F*_2,10_ = 17.198). From Stage I to Stage II, the fecal T3 levels in young FMD showed downward trends although they were not significant (*P* > 0.05). In Stage III (after weaning), the fecal T3 levels were significantly lower than in Stage I and Stage II (*P* < 0.01). The scatter plot in Fig. 5b shows that during all the three stages, changes in fecal T3 levels were positively correlated with the change of the abundance of *Bacteroides* (Spearman rank *r* = 0.858, *P* < 0.05) and *Lactobacillus* (Spearman rank *r* = 0.897, *P* < 0.05), but negatively correlated with the change of *Ruminococcaceae_UCG.005* (Spearman rank *r* = −0.594, *P* < 0.05).

### Changes of fecal IgA levels during the growth of young FMD and correlation with the intestinal microbiota

During young FMD development, there were significant differences in IgA levels between stages (Fig. 4c; *F*_2,10_ = 29.553). From Stage I to Stage II, fecal IgA levels in young FMD showed no significant differences (*P* > 0.05). After weaning (Stage III), fecal IgA levels were significantly lower than in Stages I and II (*P* < 0.01). The scatter plot in Fig. 5c shows that during all the 3 stages, changes in fecal IgA levels were positively correlated with the change of the abundance of *Bacteroides* (Spearman rank *r* = 0.816, *P* < 0.05) and *Lactobacillus* (Spearman rank *r* = 0.445, *P* < 0.05), but negatively correlated with the change of *Ruminococcaceae_UCG.005* (Spearman rank *r* = −0.792, *P* < 0.05).

## DISCUSSION

The intestinal microbiota, Cor, T3, and IgA are important parameters that characterize the growth, development and health of young mammal. Our results illustrate how the microbiota associated with diet change along with concomitant changes in the levels of Cor, T3, and IgA with young FMD growth.

Stage I is the initial stage of the establishment, development, and succession of intestinal microbiota. LEfSe analysis revealed that at phylum level, the relative abundance of Proteobacteria, Bacteroidetes in this stage were significantly higher than that in Stages II and Stage III. At the family level, Lactobacillaceae, Enterobacteriaceae, and Fusobacteriaceae in the feces of young FMD in Stage I were also significantly higher than in Stage II and Stage III, while the family Prevotellaceae significantly increased in Stage III after weaning. Since young FMD only feed on milk in Stage I, Lactobacillaceae colonization helps facilitates fermentation of the oligosaccharides in mothers’ milk (Schwab & Gänzle 2011; Thongaram *et al*. 2017). *Lactobacillus* as an important beneficial bacterium potentially could regulate the balance of intestinal microbiota, enhance immunity and resistance, and promote intestinal growth and development (Bauer *et al*. 2006; Ashraf & Shah 2014; Valeriano *et al*. 2017), which play a vital role in the early growth stage of young FMD. Bacteroidetes can decompose polysaccharides in human milk (Marcobal *et al*. 2010, 2011). Nash *et al*. (2017) showed the high abundance of Proteobacteria in feces may be related with the formation of the early immune system in infants. It should be noted, however, that in Stage I when young FMD are completely dependent on milk, cellulose‐decomposing bacteria Ruminococcaceae have also colonized the gut (Biddle *et al*. 2013). Given that the mother FMD often lick the mouth and nose of their young during this stage, the initial intestinal microbiota of the young likely come from the mother. The presence of cellulose‐decomposing bacteria in the digestive tract of the young FMD could be regarded as the pre‐adaptation of the young FMD to the subsequent feeding plants. We also noted a high abundance of *Escherichia_Shigella* within the bacterial family Enterobacteriaceae in the intestine of newborn FMD. This pathogenic bacterium may cause diarrhea and dysentery (Inoue *et al*. 2005; Konstantinov *et al*. 2006; Lalles *et al*. 2007). Highly abundant *Escherichia_Shigella* have also been detected in the feces of lactating human infants (Maltby *et al*. 2013) and piglets (Konstantinov *et al*. 2006; Kim *et al*. 2011), which may come from the maternal vagina during delivery (Azad *et al*. 2013). With the growth of young FMD, the abundance of pathogenic bacteria significantly decreased. Perhaps at the later stage of development, the establishment of beneficial bacteria may play a role in eliminating pathogenic bacteria.

Stage II is the stage when young FMD begin to gradually increase their intake of plant cellulose. Previous studies have shown that cellulose is the main source of carbon and energy for the intestinal microbiome (Lozupone *et al*. 2012; Sonnenburg & Sonnenburg 2014). Sonnenburg *et al*. (2016) found that the intestinal microbiota diversity of mice fed a low‐fiber diet remained lower than that of mice fed a high‐fiber diet. As the young FMD began eating leaves 30 days after birth and gradually increased their consumption of leaves as they grow, the relative abundance of Proteobacteria and Fusobacteria decreased, while the relative abundance of Firmicutes increased. Similarly, an inverse relationship between Firmicutes and Proteobacteria abundance was found in the milk of cows (Li *et al*. 2018). *Prevotella* and *Clostridium* are gradually increasing in this stage, which may also be due to their ability to degrade polysaccharides in plants, such as hemicellulose and xylan (Hayashi *et al*. 2007; Lamendella *et al*. 2011; Ivarsson *et al*. 2014). Also, Clostridia is an anaerobic bacterium which is specialized in the degradation of plant polysaccharides (Korpela & de Vos 2018). It is apparent that Stage II is an important period for diet to affect the composition of intestinal microbiota.

Stage III occurs after weaning. No significant differences in microbiota composition were found between Stage II and Stage III, which indicated that the establishment of intestinal microbiota had been completed before weaning. Similar studies show that during the first few weeks of early life, microbiota succession in the intestine of human infants (Favier *et al*. 2002), pigs (Inoue *et al*. 2005), and calves (Dill‐McFarland *et al*. 2017) are remarkably similar; the change of intestinal microbiota in animal development is an ongoing succession process (Backhed *et al*. 2015).

Correlation network analysis revealed that during the early succession period of intestinal microbiota community, correlations between bacteria are complex. *Bacteroides and Bifidobacterium* in Fig. 3a are positively correlated, which can digest milk polysaccharides and enhance the immune system (Marcobal *et al*. 2011; Ashraf & Shah 2014), but these two genera are negatively associated with some potential pathogens (e.g. *Escherichia_Shigella*, *Fusobacterium*) (Konstantinov *et al*. 2006; The *et al*. 2018). *Bifidobacterium* is recognized as beneficial bacteria, which can enhance host immunity, resist foreign pathogenic bacteria invasion, and promote intestinal growth and development (Bauer *et al*. 2006; Ashraf & Shah 2014; Valeriano *et al*. 2017), suggesting that the potential pathogens are replaced by beneficial bacteria in Stage I, and *Bifidobacterium* may be used as potential probiotics to maintain the intestinal health of young FMD in the future. In Stage II, some cellulose decomposing bacteria (e.g. *Ruminococcus_1*, *Ruminococcaceae_UCG.010*, *Prevotella_1*, etc.) (Biddle *et al*. 2013; Ley 2016) become dominant; there is a competitive relationship between them and milk decomposing bacteria. In Stage III, the bacterial community is more stable and distinct than stage II; the cellulose decomposing bacteria in the red module are positively correlated; they perform their functions by collaborating with each other.

The fecal Cor of young FMD remained relatively stable in Stage I and Stage II, but exhibited a sharp rise during Stage III. Some studies have shown that weaning stress can cause intestinal microbiota disorder in piglets and rats, reducing the abundance of beneficial bacteria such as *Lactobacillus*, while increasing the abundance of *Escherichia coli*; some of the strains can cause diarrhea (Yan 1993; O'Mahony *et al*. 2009). When affected by stress, the relative abundance of *Bacteroides* and *Lactobacillus* in the host intestine decreased while that of Clostridiales increased (Bailey *et al*. 2011). Although this study revealed that changes in Cor levels were negatively correlated with changes in the relative abundance of *Lactobacillus* and *Bacteroides*, the mechanism responsible for this change could not be determined. Weaning is a critical period in the breeding of young mammals. The sudden separation of mother and young are likely to imbalance intestinal microbiota, which may lead to diarrhea and decrease resistance and susceptibility to various diseases. Therefore, from the physiological perspective, weaning should be made more gradual in order to avoid the adverse effects of sudden separation of young and mother that are likely to increase Cor levels.

T3 of young FMD remained relatively stable in Stage I and Stage II, while significantly decreasing in Stage III. T3 plays a major role in metabolism and growth of young FMD. Sharma *et al*. (1985) found that T3 levels in young buffalo were highest in the first week after birth, then decreased gradually, consistent with the observations of our study. Therefore, T3 of early young FMD primarily comes from mothers’ milk. As young FMD began to eat leaves, the intake of milk decreases, and T3 level decreased after weaning. We can infer that the T3 level in the early stage of young FMD may not be affected by the intestinal microbiota or Cor. The separation of mother and young led to the increase of Cor, while T3 decreased to low level. In general, Cor varies in direct proportion to T3 (Douyon & Schteingart 2002), unlike the results of this study. This may be due to the underdeveloped thyroid gland of young FMD combined with thyroid hormone mainly coming from milk. At the same time, our results showed that the T3 level is positively correlated with *Bacteroides* and *Lactobacillus*, and negatively correlated with *Ruminococcaceae_UCG.005*. The mechanism underlying this correlation is currently unknown.

IgA of young FMD remained relatively stable in Stage I and Stage II, and then significantly decreased in Stage III. This was similar to the findings of Xi *et al*. (2010) on the fecal IgA levels in piglet from birth to weaning. Obtaining IgA with milk is crucial for the maintenance of the immune function of the intestinal tract in infants (Prentice *et al*. 1987). Studies have shown that IgA content is highest in the colostrum, and then gradually decreases (Armenio *et al*. 1980). We can see that IgA and T3 show similar trends during early development, whereby both are from milk and both gradually decrease after weaning. With the gradual development of the autoimmune system and maturation of the thyroid gland, IgA and T3 will rise again. It is worth noting that the change of IgA levels was positively correlated with the change in the relative abundance of *Lactobacillus*. Because the immune function of this beneficial bacterium is recognized (Ashraf & Shah 2014), the gradual decrease in its abundance may be another reason for early stage IgA declines in young FMD.

This study revealed that from birth to weaning, colonization of the intestinal microbiota of young FMD is a continuous and complex dynamic process. Fecal Cor levels stabilized before weaning and increased significantly after weaning, while fecal T3 and IgA levels declined gradually and continuously both before and after weaning. Infancy is an important stage for the formation of various functions of the body. Therefore, this study on the intestinal microbiota succession and dynamics of Cor, T3, and IgA will be helpful to understand the physiological and health status of young FMD. Managers should be able to monitor non‐invasively these predictable changes in these parameters in the further protection and management.

## CONFLICT OF INTEREST STATEMENT

The author(s) declare that they have no conflict of interest.

## Supporting information

Supporting information.
**Figure S1** NMDS analysis of the OTU community composition of intestinal microbiota of young FMD from birth to weaning.
**Figure S2** LEfSe analysis of intestinal microbiota of young FMD from birth to weaning.
**Figure S3** PICRUSt analysis.
