# Supplementary material for: Characterization of intestinal microbiota and fecal cortisol, T3, and IgA in forest musk deer (Moschus berezovskii) from birth to weaning
Source: Integr Zool. 2021 Jan 22;16(3):300–12. doi: 10.1111/1749-4877.12522 (PMC8248411; doi:10.1111/1749-4877.12522)
Supplement: Supplementary file 1 — Supporting information. Figure S1 NMDS analysis of the OTU community composition of intestinal microbiota of young FMD from birth to weaning. Figure S2 LEfSe analysis of intestinal microbiota of young FMD from birth to weaning. Figure S3 PICRUSt analysis. [file INZ2-16-300-s001.pdf]

SUPPLEMENTARY MATERIALS

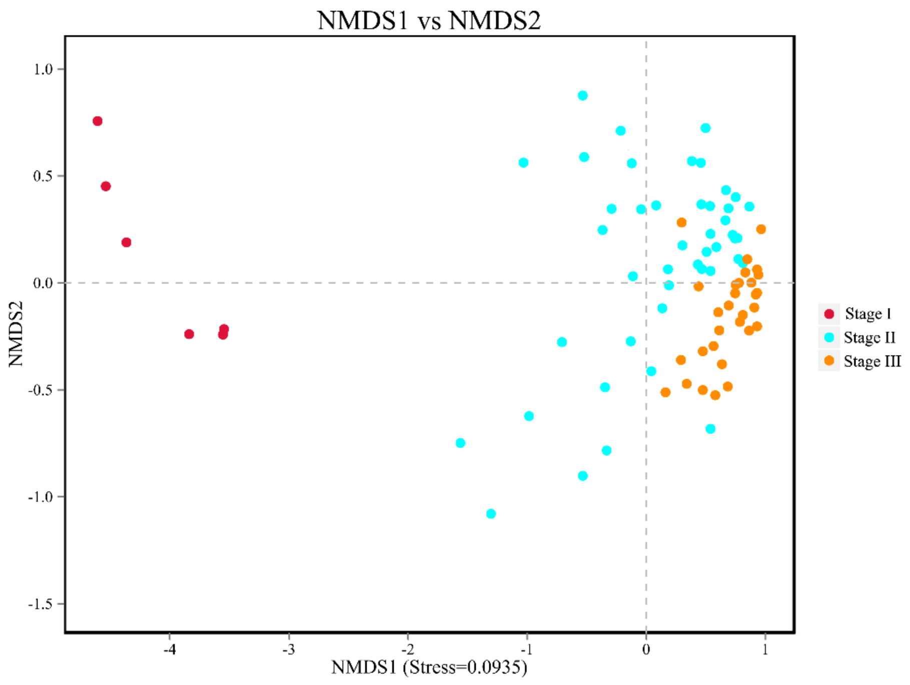

Figure S1 NMDS analysis of the OTU community composition of intestinal microbiota of young FMD from birth to weaning. Stage I: 7-10 d after birth; Stage II: 30-80 d after birth; Stage III: weaning to 30 d after weaning. The distance between points indicates the degree of difference based on unweighted Unifrac similarities of OTU composition in each sample.

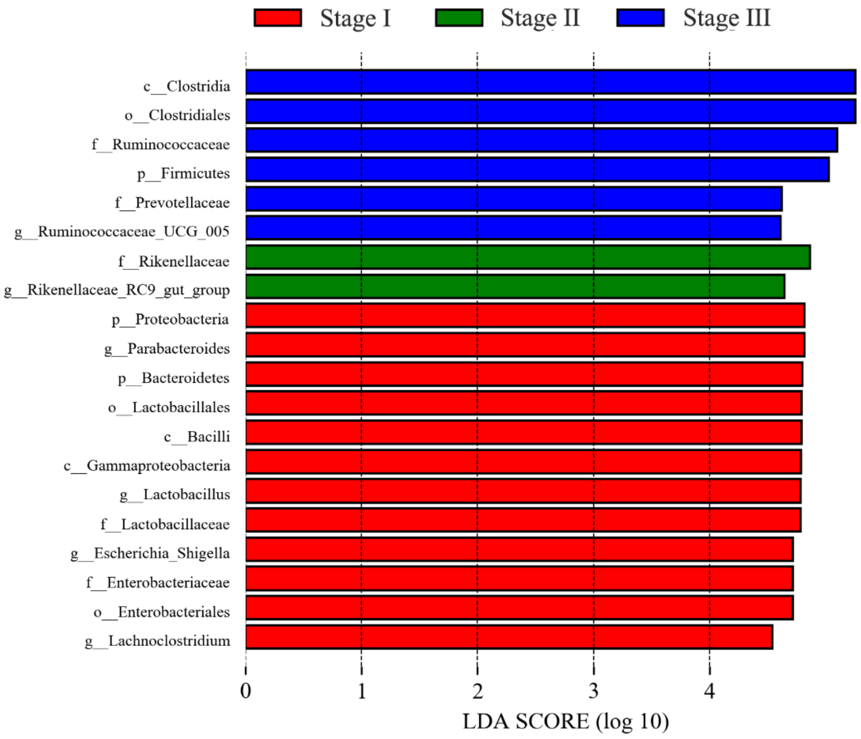

Figure S2 LEfSe analysis of intestinal microbiota of young FMD from birth to weaning. Stage I: 7-10 d after birth; Stage II: 30-80 d after birth; Stage III: weaning to 30 d after weaning. Cladogram showing the OTUs with significant differences with an LDA score greater than 4.5. Letters in front of OTU names represent the taxonomic level (p, phylum; c, class; o, order; f, family; and g, genus).

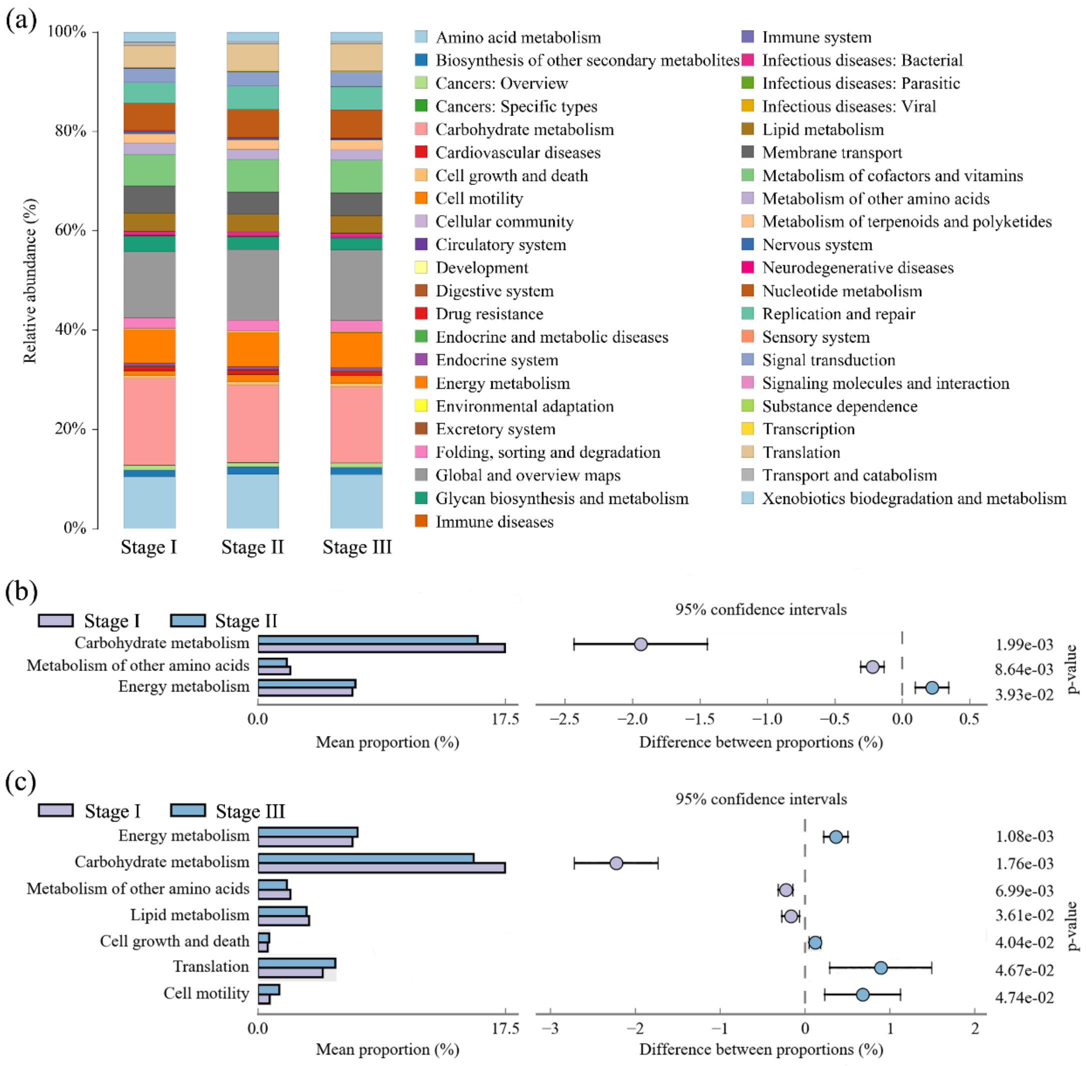

Figure S3 PICRUSt analysis. (a) KEGG metabolic pathways in the second level between three stages. The x-axis represents stages and the y-axis represents relative abundance presented as a percentage. (b) The abundance ratio of different functions between Stage I and Stage II, (c) Stage I and Stage III. The middle shows the difference between proportions of functional abundance in the 95% confidence interval, and the value at the rightmost is the *P*-value. *P* < 0.05 reflects significant differences.
